# A new, machine learning‐based approach to metastatic neuroendocrine tumors of unknown origin

**DOI:** 10.1111/jne.70134

**Published:** 2026-02-06

**Authors:** Jiaxi Lü, Tania Amin, Till Clauditz, Kira Steinkraus, Oliver Buchstab, Samuel Huber, Jakob Izbicki, Thorben Fründt, Jörg Schrader, René Werner, Rüdiger Schmitz

**Affiliations:** ^1^ Institute for Applied Medical Informatics University Medical Center Hamburg‐Eppendorf Hamburg Germany; ^2^ Institute of Computational Neuroscience University Medical Center Hamburg‐Eppendorf Hamburg Germany; ^3^ I. Department of Medicine University Medical Center Hamburg‐Eppendorf Hamburg Germany; ^4^ Deparment of Pathology University Medical Center Hamburg‐Eppendorf Hamburg Germany; ^5^ Department of General, Visceral and Thoracic Surgery University Medical Center Hamburg‐Eppendorf Hamburg Germany; ^6^ Institute of Pathology Ludwig Maximilian University Munich Germany; ^7^ Klinikum Nordfriesland Husum Germany; ^8^ Center for Biomedical Artificial Intelligence (bAIome) University Medical Center Hamburg‐Eppendorf Hamburg Germany; ^9^ Department for Interdisciplinary Endoscopy University Medical Center Hamburg‐Eppendorf Hamburg Germany

**Keywords:** cancer of unknown primary, liver metastases, machine learning, neuroendocrine tumors

## Abstract

Neuroendocrine tumors (NETs) frequently present at a metastatic stage, particularly with liver metastases. Identifying the site of the primary tumor is critical for guiding therapy but often proves difficult. Small intestine NETs are especially distinct in their prognosis and treatment. To address this challenge, we developed a novel, machine learning‐based tool to predict the site of origin—specifically small intestine or pancreas—using routine hematoxylin and eosin (H&E)‐stained slides from hepatic metastases. To avoid mislabeling in the clinically relevant scenario of any possible tumor origin, the method applies a two‐step approach with optional abstention for uncertain classifications or non‐small intestine/non‐pancreas cases. In a retrospective, clinically realistic cohort with unrestricted tumor origin, the model identified small intestine NETs with a sensitivity of 71.4% at 100% specificity and positive predictive value (PPV), and high negative predictive value. A relevant subset of pancreatic NETs can also be reliably detected (sensitivity 33.3%, specificity 94.1%, PPV 85.7%). Generalizability and robustness were rigorously validated on an external dataset using different scanners, institutions, and resection techniques. The tool is intended as an additional method where other diagnostic modalities remain inconclusive regarding the location of the primary tumor. To facilitate further research and clinical translation, all models and extracted features are publicly released.

## Graphical abstract

From HE‐stained tissue of NET hepatic metastases, a machine learning‐based tool identifies most cases with small intestine and many with pancreatic origin, while flagging all others as “uncertain” to prevent misdiagnosis. The model is validated thoroughly and made publicly available. It may impact management of metastatic NET of unknown primary.

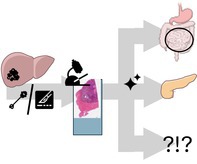

## 1 BACKGROUND

Neuroendocrine tumors (NETs) can originate in a vast range of organs and pose a complex, multidisciplinary clinical challenge.^1^ While exact numbers vary, a substantial proportion of patients are diagnosed at metastatic stage. In a cohort of over 70.000 patients with a NET of any origin, 22.0% had distant metastases at diagnosis, with the highest rate of 40.3% reported for pancreatic NETs.^2^ Meanwhile, Pape et al. reported that 69.4% of patients with gastroenteropancreatic NETs had distant metastases at initial diagnosis.^3^ NETs are often characterized by slow growth and a strong preference to metastasize to the liver^4^ and the primary tumor can be hard to identify through imaging.^5^ As a consequence, a fraction of 9–19% of all NETs are cancers of unknown primary,^6^ and hepatic metastases without known site of the primary tumor is a common and challenging scenario in NET. Depending on the site of the primary tumor, NETs demonstrate differing prognoses,^2^ molecular and genetic patterns^7^ and responses to specific therapeutic approaches, which hence plays a role in therapeutic considerations.^8^, ^9^ For instance, small‐intestine NETs typically demonstrate poor response to chemotherapy with Streptozotocin/5‐FU or the combination of Capecitabine and Temozolomide,^10^ which are the standard first‐line therapies administered to patients with metastatic progressive and/or symptomatic pancreatic NETs G1‐G2.^11^ Conversely, somatostatin analogs are the recommended first‐line treatment for NETs from the small intestine with a Ki‐67 index up to 10%. Chemotherapy is reserved for carefully selected patients with G2 disease characterized by high tumor burden, rapid progression, or elevated Ki‐67 index, as well as in the rare case of a G3 NET.^12^ Furthermore, targeted therapies aiming at mTOR pathway and multiple TKIs have been established for NETs. With the plenty of available therapeutic strategies, predictive markers for a personalized approach to treatment selection are needed.^13^

Available diagnostic approaches, including radiological and nuclear imaging, endoscopic imaging, and immunohistochemical staining, all have varying diagnostic gaps, which underlie the relatively high number of NETs of unknown primary and the long and continuing search for additional and complementary diagnostic tools.^14^ For instance, radiological and nuclear imaging techniques are limited by their spatial resolution. Wang et al. show that CT and somatostatin receptor (SSTR) scintigraphy demonstrate low sensitivity for detecting NET primaries located in the gastrointestinal tract due to their small size, as revealed after surgical exploration.^5^ The size of the primary, besides low SSTR expression or adjacency to sites of physiological uptake, can also be relevant limitations to somatostatin receptor PET/CT imaging.^15^ Immunohistochemical staining for localizing the primary tumor typically includes CDX2 for midgut, TTF1 for lung, and PAX8 or ISLET1 for pancreas NETs as immunohistochemical markers.^6^ Despite the diagnostic value of immunohistochemistry (IHC), it is still limited by the nonspecific expression of these proteins in other organs and the lack of expression in some instances. In the absence of expression of CDX2, TTF1, and PAX8/ISLET1, referred to as the triple‐negative pattern,^6^ reliable conclusions about the primary tumor location cannot be drawn.

Given the relevance of the primary tumor site for clinical management, there is a long^16^ and sustained^17^ clinical need for and interest in *additional and complementary* biomarkers and diagnostic means to identify the primary tumor site, guide further diagnostics and, if remaining inconclusive, perhaps also (empiric) therapy. This need is further underscored by the fact that validated predictive molecular biomarkers for gastroenteropancreatic NETs are still lacking, and treatment personalization currently relies mainly on clinical and pathological assessment.^13^

Considering the substantial advancements in machine learning (ML) in general and also in NET in particular,^18^, ^19^ including recent works for the improved detection of small NET lesions in CT imaging^20^ and for the discrimination between neuroendocrine and colorectal cancer in surgical specimens,^21^ ML holds the potential to address this need. This study examines the potential diagnostic value of an ML model to predict the site of the primary tumor from hematoxylin & eosin (H&E) stained whole‐slide images (WSIs) of hepatic metastases. Characteristic morphological features for NETs of the small intestine were described by Williams and Sandler.^22^ Redemann et al. demonstrated that, given digitized H&E‐stained slides of a NET primary tumor, ML can achieve comparable results to IHC in determining the surrounding host organ from patches of the primary tumor itself despite only having a low two‐digit amount of WSIs for each site of origin available as training data.^23^ However, the examined scenario does not resemble a real scenario with a NET of unknown primary, in which only histological material of metastases, not the primary site, is available.

The present study addresses this by utilizing the, to the best of the authors' knowledge, currently largest dataset of NET liver metastases WSIs. Based on WSIs of NET liver metastases, it will be shown that a lightweight ML model, resilient to overfitting, can accurately distinguish between metastases originating from the pancreas and small intestine. Additionally, in a more realistic clinical scenario where metastases can potentially arise from any source, our model demonstrates a high level of accuracy in identifying metastases from the small intestine. This capability enables the prompt and precise identification of small intestine origin NETs in real‐world diagnostic scenarios. Model generalizability is demonstrated on an external test set, which, as opposed to the training set, consists of preferably biopsies rather than full resection specimens.

## 2 METHODS

### 2.1 Data collection

#### 2.1.1 UKE dataset

Clinical files of patients with known liver metastasized NETs treated at the University Medical Center Hamburg‐Eppendorf (UKE) from 01.01.2010 to 31.12.2020 were retrospectively explored to identify all NET patients with hematoxylin and eosin (H&E) stained slides of liver resections or liver biopsies containing tumor material available in the hospital's archives. We included only patients whose primary tumor sites were confirmed through pathology or conclusive imaging. 119 patients fulfilled these criteria. 20 out of these 119 patients had to be excluded due to insufficient quality of the slides because of artifacts or not enough tumor material visible on available slides. The remaining 99 patients defined the UKE dataset. The tumors of these patients originated from the following sites (Table 1): small intestine (51), pancreas (40), kidney (1), rectum (3), and lung (4). For each patient, up to three H&E‐stained slides for each liver resection or biopsy were collected from the hospital's archive. This resulted in 270 slides (219 from resection and 51 biopsy specimens). The slides were then digitized using a 3DHISTECH Pannoramic 1000 scanner at 80× magnification.

**TABLE 1:** Characteristics of the UKE and the external dataset.

		UKE dataset	External dataset
Age at initial diagnosis	Mean ± standard deviation (years)	56.8 ± 13.9	50.2 ± 13.2
Sex	m	59	15
	f	40	11
Primary tumor site	Pancreas	40	9
	Small intestine	51	14
	Rectum	3	1
	Lung	4	2
	Kidney	1	0

#### 2.1.2 External dataset

An external test dataset was collected for unbiased model validation. This dataset includes collected samples from 15 patients treated at the Klinikum of the Ludwig Maximilian University (LMU) of Munich. The entire process of slide preparation and slide digitization was also performed externally at the Institute of Pathology of LMU. To supplement this dataset, the 195 patients of the original 314 UKE patient cohort that were excluded in the first step (see above) were examined to identify patients with liver metastasized NET and a confirmed primary tumor site who had undergone biopsy or resection of their liver metastases before initial presentation at the UKE. 21 eligible candidates were identified for inclusion in the dataset. For 15 of these, tissue slides were requested by informed consent of the patients from the respective external clinic or laboratory where the tissue sample had been processed. Three patients have been excluded due to delayed or no response by the external institution. One more patient had to be omitted from this study due to the only available slide not containing enough visible tumor material.

As a result, our complete external validation dataset comprised 37 slides of 20 biopsies and six resections, obtained from 26 patients. Among these patients, 9 had primary tumors in the small intestine, 14 had primary tumors originating from the pancreas, 2 from the lung, and 1 from the rectum (Table 1).

The slides of this dataset were digitized using a Leica Aperio GT450 (Munich samples) and a Leica Aperio AT 2 scanner (non‐Munich samples), each at 20× magnification. Note that we deliberately employed different scanners than for the internal data to make the external testing scenario strict and thorough. In the same sense, it should also be noted that the external test set differs from the internal data in that the external data is mostly from biopsies rather than resections.

### 2.2 Data preparation

**FIGURE 1:**
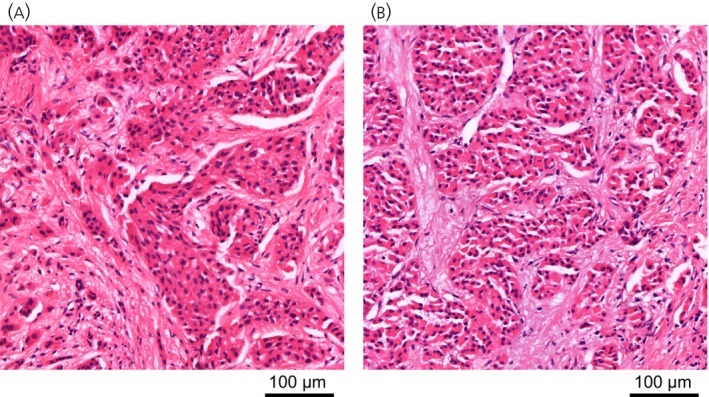
Liver metastases patches. Patches from the UKE dataset of size 4096 × 4096 pixels (equivalent to 497 × 497 μm) depicting liver metastases of small intestine (A) and pancreatic (B) origin.

Tumor regions and areas with visible artifacts were manually segmented using QuPath v0.3.^24^ For each patient, 100 patches were randomly chosen, with dimensions of 4096 × 4096 pixels for the UKE dataset and, to account for the different image resolution, 2048 × 2048 pixels for the external dataset. Exemplary patches from the UKE dataset are shown in Figure 1. Since a patient may have undergone multiple biopsies/resections, each with multiple available H&E slides, the 100 patches were evenly distributed across all biopsies/resections of a patient and subsequently across each slide within the respective biopsy/resection. This ensured that each patient's slide contributed equally during ML model training. Patches were randomly selected from tumor regions without notable artifacts. Subsequently, the patches were resized to dimensions of 224 × 224 pixels, and the color was standardized with a mean of [0.485, 0.456, 0.406] and a standard deviation of [0.229, 0.224, 0.225] across the three RGB color channels. Patches in this standardized format served as input for the ML models.

### 2.3 Experiments

For automated identification of the NET site of origin, we propose a two‐step approach: Use of a binary classifier for the most common sites of origin, that is, small intestine and pancreas, followed by a second step to identify outliers which cannot be reliably attributed to either of the common origins.

This is justified by two reasons: First, the rarity of NET liver metastases originating from the rectum, lung, or kidney renders direct training of a ML model for multi‐class classification impractical. Second, the two‐step approach is tailored to the clinical scenario of application: From the general population, narrow down the potential sites of origin as far as possible and tell the user where no decision can be deduced.

#### 2.3.1 Binary classification small intestine vs. pancreatic origin as a subtask of the clinical problem

To perform the first classification task, first, a patch‐level classifier was trained on 100 randomly selected patches for each training patient drawn from the manually segmented tumor areas (cf. Section 2.2). Due to the limited number of patients, we employed a transfer learning approach for the patch‐level classifier. For the feature extractor, we used a pretrained standard convolutional neural net (ResNet50). Here, feature extraction refers to the process of transforming the image data into numerical representations, called embedding vectors, through the pretrained ResNet50, thereby leveraging its existing knowledge learned during pretraining to generate meaningful representations. As we follow a transfer learning approach and leave the weights of the feature extractor untouched, the choice of the pretraining dataset can be potentially crucial and should be examined. We evaluated three different variants: first, an ImageNet‐pretrained ResNet50; second and third, to obtain potentially more representative image patch features, two additional ResNet50 models that had been pretrained on histopathological data were used: “MTDP”^25^ and “RetCCL.”^26^ Morment et al. used a multi‐task ResNet50‐based architecture for image classification. They gathered 22 different publicly available histopathological datasets with a corresponding segmentation, detection, or classification task each. Wang et al. employed a self‐supervised contrastive learning approach to train a feature extractor using the cancer genome atlas (TCGA) (https://www.cancer.gov/about‐nci/organization/ccg/research/structural‐genomics/tcga, accessed 16 March 2025) and the pathology AI platform (PAIP) dataset,^27^ both extensive collections of histopathological images from a wide variety of organs, for a subsequent histopathological image retrieval task.

The input of the fully connected layer of the ResNets was used as the image patch representation, that is, the embedding vector, and served as the input for the ML patch classifier. For classification, the standard scikit‐learn (version 1.1.2) implementations for a support vector machine (SVM) with a linear and a radial basis function (RBF) kernel as well as the git scikit‐learn logistic regression (LR) model were used. No hyperparameter optimization was performed with all parameters left as their defaults (SVM: inverse regularization strength *c* = 1.0, degree 3, gamma = “auto”; LR: L2 penalty term, *c* = 1.0, default solver, maximum 100 iterations; cf. https://github.com/IPMI-ICNS-UKE/NET_CUP/ for a detailed reference).

Ultimately, for patient‐level inference, a majority vote is performed on the classification results of 100 patches randomly drawn from the tumor areas within the slides of the test patient. For evaluation, a leave‐one‐out cross‐validation is conducted.

#### 2.3.2 Uncertainty measure to extend the binary classifier for applicability to tumors of arbitrary origin

Non‐small intestine‐non‐pancreatic metastasized NETs are rare. Nevertheless, in a real clinical scenario of metastasized NET of unknown primary, the origin cannot be assumed to be restricted to the two most common primary sites, namely small intestine and pancreas. We refer to such cases collectively as “other origin.”

As these stem from a multipolicy of different anatomical sites, the corresponding feature distributions cannot be assumed to be homogeneous. Therefore, inclusion of “other origin[s]” as an additional output label of a single‐step classifier is infeasible given the available data.

To achieve a clinically applicable yet robust classification pipeline, we propose to employ a *binary* classifier trained to distinguish between pancreatic and small‐intestine NETs. Rather than retraining a model with additional output labels, we use its intrinsic decision function to identify cases likely to originate from other, unmodeled sources by treating such predictions as “uncertain.”

##### Classifier‐based uncertainty estimation

**FIGURE 2:**
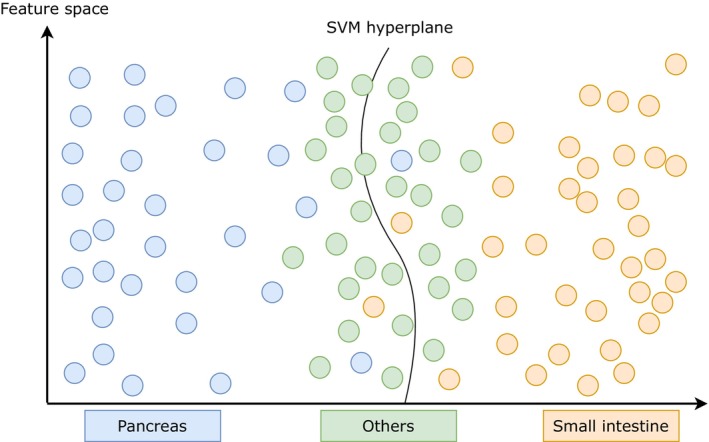
Support vector machine (SVM) hyperplane within the feature space that is used as an input for the classifier. Each dot symbolizes the feature vector of a patch. The SVM hyperplane is trained as the optimal solution for effectively distinguishing between pancreatic and small intestine patches. The absolute value of the distance from the hyperplane reflects the overall certainty, while the sign of the signed distance indicates the most probable class. Atypical pancreatic NETs, atypical small intestine NETs, and NETs from other origins are found in closer proximity to the SVM hyperplane.

We hypothesize that feature vectors associated with patches of NETs from other origins lack the characteristic traits of pancreatic or small intestine NETs. Consequently, they will lie closer to the classifier decision boundary in the latent feature space, with the absolute value of the distance to the hyperplane a measure of uncertainty for the classification of an individual patch (cf. Figure 2), while the sign of the signed distance indicates to which class the patch belongs. At the patient level, uncertainty can be aggregated by summing all signed distances from the SVM hyperplane across patches belonging to the same patient.

##### Determination of uncertainty thresholds

This algorithm requires determination of two thresholds: Any case for which the summed signed distance lies between these two is labeled “uncertain.” These are two parameters of the algorithm that need to be optimized on the training set.

With 60% of the patients from the UKE dataset with either pancreatic or small intestine origin already used for training of the patch‐level machine learning classifier, as described in Section 2.3.1, the remaining 40% of the UKE training patients with pancreatic and small intestine NETs as well as another o=8 UKE dataset patients with another NET origin are used to determine the uncertainty thresholds. For these cases, the sum of signed patch distances to the SVM hyperplane for each sample were computed. Lower and upper thresholds can then be determined for each possible value $i\in \left\{0,1,\ldots ,o\right\}$ such that the sums of signed SVM distances of exactly i of the o patients with NETs of other origin were within these thresholds while the number of patients with pancreatic and small intestine NETs in that interval was minimized. The parameter *c* = i/o is exactly the portion of the cases of other origin (correctly) labeled “uncertain” with respect to the binary classification, hence a confidence parameter.

**FIGURE 3:**
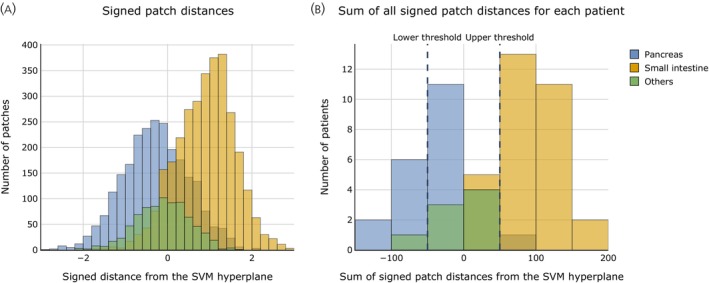
Signed patch‐to‐hyperplane distances and derivation of the thresholds for the uncertainty class. (A) Signed distances to the SVM hyperplane (radial basis function kernel) for individual tissue patches from NETs of pancreatic, small intestine, and other origin. The SVM was trained on 40% of all pancreas and small intestine patients, while the rest of the UKE dataset was used to calculate distances to the hyperplane for 100 patches per patient. (B) Sum of the signed patch‐to‐hyperplane distances for 100 patches per patient. The portion of cases of other origin that shall correctly be classified as “uncertain” with respect to pancreatic versus small intestine origin determines the thresholds for this class. Here, the thresholds for a confidence parameter of c=i/o=7/8=0.875 are shown. Any case with a summed signed patch‐to‐hyperplane distance between these two thresholds will be classified as “uncertain.”

This is followed in Figure 3, which depicts the distribution of signed patch distances (A) and the total sum of these distances for each patient (B). A noticeable overlap of signed distances for patches of NETs from different origins appears near the decision boundary in Figure 3A and persists when aggregated at the patient level in Figure 3B. In Figure 3B, for a chosen confidence parameter, defined by the proportion of patients with a NET of other origin included within the thresholds, the thresholds can be derived on the x‐axis.

Both the patch classifier as well as the classification thresholds are optimized solely on the internal UKE dataset. Performance on that internal UKE dataset is evaluated by leave‐one‐out cross‐validation.

All external data is thoroughly left out for any step of model training, classifier training, or threshold choice, hence entirely left for a thorough external testing.

## 3 RESULTS

The full clinical problem of identification of the primary origin in a general population corresponds to classification into an arbitrary number of rare and heterogeneous classes. This is obviously hard. Therefore, as said, we approach the problem over two steps: (i) Identification of the two most common sites of origin, pancreas and small intestine, followed by (ii) a mechanism to rule out those cases which cannot safely be attributed to either of them, hopefully containing all cases originating from other organs.

### 3.1 Binary classification small intestine vs. pancreatic origin as a subtask of the clinical problem

The results for the binary classification task are presented in Table 2. The classifier technically works on individual tissue patches (left three columns). For each patient, 100 patches are randomly sampled from the tumor area, and the patient is assigned to either the small intestine or the pancreatic origin class depending on how the majority of the individual patches are classified (right three columns).

**TABLE 2:** Classification of pancreas and small intestine origin in a dataset with only these two origins.

Feature extractor/ML model	Classification of individual tissue patches			Classification at patient level		
	SVM RBF	SVM Linear	LR	SVM RBF	SVM Linear	LR
ImageNet	0.778	0.753	0.750	0.890	0.890	0.868
MTDP	0.792	0.779	0.782	0.912	0.912	0.890
RetCCL	**0.832**	0.799	0.802	**0.934**	0.923	0.901

*Note*: The table reports the average accuracies for the binary classification task using all combinations of three different pretrained ResNet50s as feature extractors and three different machine learning (ML) models that were applied as patch‐level classifiers on the patients with NETs of pancreatic and small intestine origin (*n* = 91): a support vector machine (SVM) with a radial basis function (RBF) kernel, an SVM with a linear kernel, and a logistic regression (LR) model. Patch‐level accuracies are computed based on the classification results of 100 randomly selected patches per patient. Patient‐level classification is obtained by majority voting (cf. Section 2.3.1). The best feature extractor/classifier combinations are highlighted in bold.

Regardless of the concrete choice of ML classifier and feature extractor, a consistently high classification accuracy was achieved. The best accuracy of 93.4% was achieved through an SVM with an RBF kernel based on the histopathology‐pretrained RetCCL feature extractor. Using a standard ImageNet‐pretrained model for feature extraction and linear classifiers such as an SVM with a linear kernel or LR, the accuracy only marginally decreased.

To examine model robustness with respect to differing biopsy and resection techniques, tissue preparation, staining as well as scanning procedures, the identical task is performed on an external test dataset. The classifier is trained solely on patients from the UKE dataset with either pancreatic or small‐intestine tumor origin. The results are shown in Table 3. The highest accuracy of 84.9% on patch level is close to the in‐house counterpart, and a peak accuracy of 8/9 on patient level is attained when employing a linear SVM with the MTDP or RetCCL feature extractor.

**TABLE 3:** Accuracies for the binary classification task between pancreas and small intestine on the external test dataset (*n* = 23).

Feature extractor/ML model	Classification at patch level			Classification at patient level		
	SVM RBF	SVM Linear	LR	SVM RBF	SVM Linear	LR
ImageNet	0.608	0.583	0.595	0.652	0.652	0.652
MTDP	0.685	0.741	0.732	0.739	**0.870**	0.826
RetCCL	0.763	0.753	**0.771**	0.826	0.783	0.783

*Note*: The performance was evaluated using three different pretrained convolutional neural networks as feature extractors and three different machine learning (ML) models as patch‐level classifiers: a support vector machine (SVM) with a radial basis function (RBF) kernel, an SVM with a linear kernel, and a logistic regression (LR) model. The training was performed using only the UKE dataset. The best accuracies are highlighted in bold.

It should be noted that the external dataset almost exclusively consists of biopsies, while the UKE dataset, on which the patch‐classifier was trained, mainly consists of resection specimens.

### 3.2 Uncertainty measure to extend the binary classifier for applicability to tumors of arbitrary origin

We have so far seen that, for a set of tumors solely of pancreatic and small intestine origin, individual cases can accurately be assigned either of the two classes. However, this falls short of the clinical problem where the origin can obviously not be narrowed down to these two locations. Therefore, it is necessary to handle the existence of other tumor origins.

Based on the results for the internal dataset in Section 3.1, we selected a RetCCL feature extractor and an SVM with an RBF kernel as classifier for the subsequent experiments. Note that Table 3 would suggest that, on the test set, another configuration may achieve a marginal improvement over the best configuration as chosen from the training set. However, for the sake of a rigorous treatment of training and testing data, we proceed with the best configuration as identified on solely the training data.

Aiming at the clinically realistic scenario of classifying tumor origins into small intestine, pancreatic, and others by use of a binary classifier, the signed distances from the SVM decision boundary are used as a measure of certainty.

**FIGURE 4:**
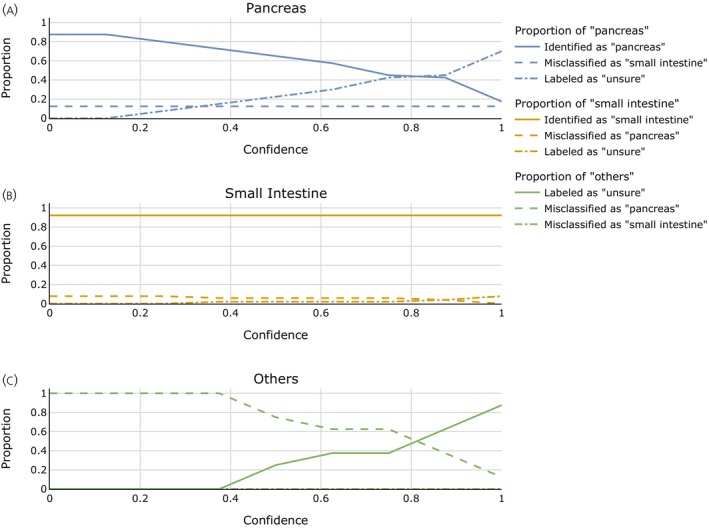
Classification performance of the extended model on the UKE dataset depending on the chosen confidence parameter. The graphs depict the portion of patients with pancreatic (A) and small intestine (B) origin who were correctly classified for the respective category (solid line), misclassified for the other origin (dashed line), or labeled as “uncertain” (dashed‐dotted line) on the UKE dataset. Panel (C) shows how tumors of another origin than pancreas or small intestine are classified. Results are obtained by leave‐one‐out validation for a varying confidence parameter i/o. One sees that while misclassifications between pancreatic and small intestine origin are rare, the choice of a higher confidence parameter is necessary to avoid misclassification of NETs of other origin as being from pancreatic origin.

To acknowledge the existence of another, heterogenous class of tumors (other origin) and still use a binary classifier, a confidence threshold is introduced as described in Section 2.3.2. The aim is to assign only “confidently” pancreas or small intestine classified cases these categories while rejecting classification of uncertain cases. The results for all confidence parameters i/o are shown in Figure 4. Table 4 shows the confusion matrices for confidence 0 (A), 0.875 (B), and 1.0 (C). The panels (D)–(F) are the corresponding results when testing on external data.

**TABLE 4:** Classification results for the extended model on the UKE dataset with confidence 0 (A), 0.875 (B), and 1.0 (C) and on the external dataset for the same confidence levels of 0 (D), 0.875 (E, bold), and 1.0 (F).

		UKE dataset			External dataset			
True origin/predicted origin		Pancreas	SI	Uncertain	Pancreas	SI	Uncertain	
A	Pancreas	35	5	0	9	0	0	D
	SI	4	47	0	4	10	0	
	Others	8	0	0	3	0	0	
B	Pancreas	17	5	18	**3**	**0**	**6**	E
	SI	2	47	2	**1**	**10**	**3**	
	Others	3	0	5	**0**	**0**	**3**	
C	Pancreas	7	5	28	3	0	6	F
	SI	0	47	4	1	10	3	
	Others	1	0	7	0	0	3	

*Note*: For the UKE dataset, results are obtained using leave‐one‐out cross‐validation with a subset of the training patients with NET of pancreatic or small intestine (SI) origin used for training the support vector machine while the remaining training patients are used for determining the thresholds as outlined in Section 2.3.2. For testing on the external dataset, the complete UKE dataset was utilized for training the support vector machine and determining the thresholds.

Setting the confidence parameter to 0 results in the same classification as in Section 3.1 for patients with NETs of pancreatic or small intestine origin, while every patient with a NET of another origin is categorized as either pancreas or small intestine (Table 4A).

When increasing the confidence parameter to 1.0, the rate of misclassification for patients with NETs of pancreatic or small intestine origin nearly diminishes while the sensitivity for the detection of small intestine NETs remains at 92.2% (positive predictive value, PPV: 90.4%, specificity 89.6%). Also, pancreatic cases can be identified with high confidence (specificity 91.5% or 98.3% at confidence 0.875 or 1.0, respectively; PPV 77.3% or 87.5% at the same confidence levels). Though, this applies to a lesser portion of the cases with only a minority being labeled as pancreatic by this tool and the majority being refused to classify (labeled “uncertain”). This is reflected by sensitivities of 42.5% and 17.5% at confidence levels of 0.875 and 1.0, respectively. The portion of pancreatic origin tumors being identified must be traded for the confidence to correctly identify non‐pancreatic‐non‐small intestine origin as “uncertain.” However, even if requiring a high confidence, a relevant portion of the tumors from pancreatic origin is extracted from the general population. Combined, 64.4% (54.4%) of the cases of a priori unknown origin are correctly labeled as either of small intestine or pancreatic origin at a confidence level of 0.875 (1.0). With the clinical scenario in mind, a confidence level of 0.875 is therefore chosen for the external testing.

Table 4E summarizes the final model performance on the external test set. When applied to a general cohort with unrestricted tumor origins, our pipeline achieved a sensitivity of 71.4% for small‐intestine NETs and 33.3% for pancreatic NETs, while maintaining a high specificity of 100% and 94.1%, respectively. Importantly, all NETs originating from sites other than the pancreas or small intestine were correctly rejected from classification into these two categories.

When re‐iterating the analysis on a subgroup of *N* = 7 cases from the test set where the origin of the primary tumor was indeed unknown at the time of surgery, the pipeline achieved a consistent performance (66.7% and 50% sensitivity of small‐intestine and pancreatic NETs, respectively, both at 100% specificity).

These results demonstrate the model's capability to accurately identify small‐intestine NETs and a relevant proportion of pancreatic NETs within a heterogeneous, clinically realistic population. Only a minimal number of misclassifications occurred, even when tumors of arbitrary origin were included. Overall, the tool fulfills its intended purpose: to effectively narrow down the large pool of metastatic NET cases with an a priori unknown primary by *reliably recognizing those of clear pancreatic or small‐intestine origin*.

**FIGURE 5:**
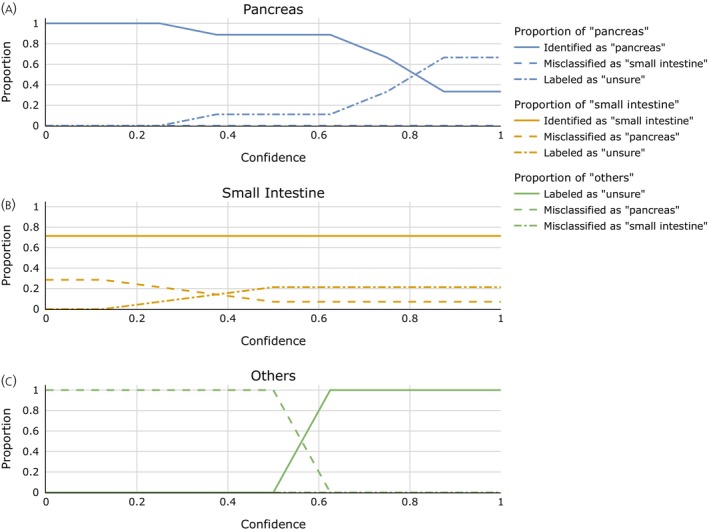
Classification performance of the extended model on the external dataset depending on the chosen confidence parameter. The findings on label‐wise accuracies for the detection of pancreatic or small intestine origin were verified on the external test set, while still modeling the clinically relevant scenario of an a priori unknown origin. Solely the UKE dataset was used for training the classifier and determining the thresholds based on the sum of signed distances from the support vector machine hyperplane. One again finds that identification of small intestine‐cases from the general population is possible with good precision. For pancreatic origin, a high identification rate is achieved at lower confidence levels. When applying a higher confidence parameter, the rate decreases, with only a limited number of cases identified. However, misclassifications as small intestine origin are nonexistent, with the pancreatic cases instead labeled as “uncertain” at higher confidence levels. Altogether, the clinical value and applicability of our approach: From the general population of all NET origins, a high portion of small intestine and a relevant number of pancreatic cases can be identified with high confidence, all others being labeled as “uncertain” (rather than being misclassified).

In closing, let us examine the effect of the uncertainty thresholding on the external test set. To this end, Figure 5 and panels D and F of Table 4 depict the results at other confidence levels. Consistent with the results on the UKE dataset, one can see that with a higher confidence level, misclassifications of other origins as small intestine or pancreatic are suppressed. Also, following the results on the UKE dataset alone, this primarily comes at the expense of fewer pancreatic NETs being identified as such. Interestingly, also misclassifications of small intestine NETs as pancreatic NETs, which have rarely been observed on the UKE dataset alone at all but when testing the pure classifier on external data, are curated with the confidence thresholds.

## 4 DISCUSSION

This study pioneers a machine learning (ML)‐based approach for identifying the primary tumor site in neuroendocrine tumors (NETs) metastatic to the liver and demonstrates its clinical feasibility. It demonstrates that machine learning can assist in identifying the primary tumor site in NET liver metastases, serving as an additional diagnostic tool to reduce cases of NETs of unknown origin when conventional imaging and immunohistochemistry (IHC) fail to provide definitive answers. To our knowledge, this study leverages the largest curated dataset of digitized H&E‐stained whole‐slide images from NET liver metastases, ensuring a robust foundation for model training.

This study demonstrates how machine learning can assist in identifying the primary tumor site in NET liver metastases. Small intestine NETs are detected with 71.4% sensitivity, 100.0% PPV, and 100.0% specificity, while pancreatic NETs are identified with 94.1% specificity, though with lower sensitivity (33.3%). The introduction of an “uncertain” category ensures that rare or unrepresented primary sites are not misclassified, preserving diagnostic accuracy. This feature makes the strictest confidence level particularly suitable for clinical use, as it enables reliable classification while minimizing the risk of clinically consequential misclassifications.

Biases of training data and differences between training and real‐world data (e.g., different resection and workup procedures, different labs and scanners) may pose important limitations for the actual clinical applicability of machine learning‐based approaches. This makes rigorous testing pivotal. We therefore collected an external cohort for testing, mainly consisting of samples from an unseen resection technique and scanned on another scanner. The independent external dataset confirms its generalizability across variations in staining, slide‐scanning technologies, and even resection/biopsy techniques. It should be noted that the external dataset almost exclusively consists of biopsies, while the UKE dataset, on which the patch‐classifier was trained, mainly consists of resection specimens.

The methodology is highly accessible, integrating seamlessly with standard digital pathology workflows. It can be readily applied to existing H&E slides of NET liver metastases, including biopsies containing as little as 1 cm of tumor tissue. Only a single slide from a metastasis is required. Any commercial whole‐slide scanner can be used, as 20× magnification is sufficient since scanned image patches with a real‐world size of 497 × 497 μm are downsized to 224 × 224 pixels as input for the ResNet.

This study has important limitations: While the model effectively reduces diagnostic uncertainty and identifies small intestine NETs with high confidence, its lower sensitivity for pancreatic NETs leaves a considerable number of cases labeled as “uncertain.” The model's reliance on histopathological features alone means it does not incorporate molecular or genetic markers that could further refine classification in the future. Importantly, the dataset includes few cases from rare origins of NET liver metastases such as lung, rectum, and kidney, restricting conclusions about the model's performance in these subgroups. Although validated externally, broader multi‐center studies will be needed to confirm generalizability across diverse clinical settings. Finally, the number of cases with an unknown primary origin at the time of hepatic surgery which was later revealed is limited in a retrospective setting as in this study. Therefore, the model performance in cases in which the search for the primary origin remained inconclusive and its benefit in clinical practice need to be evaluated in prospective studies.

For future work, it will furthermore be tempting to explore how (machine learning‐based analysis of) tumor morphology correlates with biological behavior and therapy response. Identifying morphologically distinct subtypes—such as “small intestine‐like” or “pancreatic‐like” NETs—could refine treatment selection beyond anatomical classification. Expanding the dataset to include rare NET entities and integrating molecular and genetic data may further enhance the model's predictive accuracy. Ultimately, one may hypothesize whether ML‐based morphological classification of metastasized NETs of unknown primary site may not only provide actionable support for the choice for targeted follow‐up investigations, as this study suggests, but also improve calculated therapy selection in cases where the primary tumor origin remains unknown.

In conclusion, this study demonstrates that machine learning applied to standard H&E‐stained slides of NET liver metastases can reliably identify small intestine and a relevant portion of pancreatic origin cases from a population of liver‐metastasized NETs of any origin. Incorporation of an uncertainty measure and an “uncertain” category to reject ambiguous cases was key to achieving promising clinical feasibility in the heterogenous cohort with NETs of any origin. The tool fulfills its intended purpose: to effectively narrow down the pool of metastatic NET cases with an a priori unknown primary by *reliably recognizing (“sorting out”) those of clear pancreatic or small‐intestine origin*. Our results highlight the potential of ML‐based image analysis to complement existing diagnostics, particularly in cases of NETs of unknown primary when conventional diagnostic approaches are inconclusive. Future work should extend the approach to rarer NET origins, include molecular markers and evaluate integration into clinical workflows.

## AUTHOR CONTRIBUTIONS

**Jiaxi Lü**: Data curation, formal analysis, investigation, methodology, software, visualization, writing—original draft, writing—review and editing. **Tania Amin**: Investigation, resources. **Till Clauditz**: Investigation, data curation, validation. **Kira Steinkraus**: Data curation, investigation, formal analysis. **Oliver Buchstab**: Investigation, resources, validation. **Samuel Huber**: Resources, project administration. **Jakob Izbicki**: Resources, data curation. **Thorben Fründt**: Resources, data curation. **Jörg Schrader**: Conceptualization, resources, supervision, writing—review and editing. **René Werner**: Conceptualization, methodology, project administration, resources, supervision, visualization, writing—review and editing. **Rüdiger Schmitz**: Conceptualization, investigation, methodology, software, supervision, visualization, writing—original draft, writing—review and editing.

## CONFLICT OF INTEREST STATEMENT

All authors declare no financial or non‐financial competing interests. Rüdiger Schmitz is a shareholder of casuu GmbH (https://casuu.com), which operates in the medical and professional language education technology space. This work is entirely unrelated to the company's products or services.

## ETHICS STATEMENT

This study adhered to the principles outlined in the Declaration of Helsinki and received approval from the local ethics committee (Ethics Commission Hamburg) under the number 2023‐101034‐BO‐ff.

## Data Availability

The datasets used and/or analyzed are available from the corresponding author upon reasonable request. The extracted feature vectors for training the ML models as well as trained models are available under https://github.com/IPMI-ICNS-UKE/NET_CUP/ and free to use for research purposes, provided citation of this work (CC BY‐NC‐SA 4.0).

## References

[1] Oronsky B , Ma PC , Morgensztern D , Carter CA . Nothing but NET: a review of neuroendocrine tumors and carcinomas. Neoplasia. 2017;19(12):991‐1002. doi:10.1016/j.neo.2017.09.002 29091800 PMC5678742

[2] Man D , Wu J , Shen Z , Zhu X . Prognosis of patients with neuroendocrine tumor: a SEER database analysis. Cancer Manag Res. 2018;10:5629‐5638. doi:10.2147/CMAR.S174907 30519109 PMC6239108

[3] Pape UF , Berndt U , Müller‐Nordhorn J , et al. Prognostic factors of long‐term outcome in gastroenteropancreatic neuroendocrine tumours. Endocr Relat Cancer. 2008;15(4):1083‐1097. doi:10.1677/ERC-08-0017 18603570

[4] Riihimäki M , Hemminki A , Sundquist K , Sundquist J , Hemminki K . The epidemiology of metastases in neuroendocrine tumors. Int J Cancer. 2016;139(12):2679‐2686. doi:10.1002/ijc.30400 27553864

[5] Wang SC , Parekh JR , Zuraek MB , et al. Identification of unknown primary tumors in patients with neuroendocrine liver metastases. Arch Surg. 2010;145(3):276‐280. doi:10.1001/archsurg.2010.10 20231629

[6] Bellizzi AM . Assigning site of origin in metastatic neuroendocrine neoplasms: a clinically significant application of diagnostic immunohistochemistry. Adv Anat Pathol. 2013;20(5):285‐314. doi:10.1097/PAP.0b013e3182a2dc67 23939147

[7] Walenkamp A , Crespo G , Fierro Maya F , et al. Hallmarks of gastrointestinal neuroendocrine tumours: implications for treatment. Endocr Relat Cancer. 2014;21(6):R445‐R460. doi:10.1530/ERC-14-0106 25296914

[8] Alexandraki K , Angelousi A , Boutzios G , Kyriakopoulos G , Rontogianni D , Kaltsas G . Management of neuroendocrine tumors of unknown primary. Rev Endocr Metab Disord. 2017;18(4):423‐431. doi:10.1007/s11154-017-9437-9 29199361

[9] Del Rivero J , Perez K , Kennedy EB , et al. Systemic therapy for tumor control in metastatic well‐differentiated gastroenteropancreatic neuroendocrine tumors: ASCO guideline. J Clin Oncol. 2023;41(32):5049‐5067. doi:10.1200/JCO.23.01529 37774329

[10] Auernhammer CJ , Spitzweg C , Angele MK , et al. Advanced neuroendocrine tumours of the small intestine and pancreas: clinical developments, controversies, and future strategies. Lancet Diabetes Endocrinol. 2018;6(5):404‐415. doi:10.1016/S2213-8587(17)30401-1 29229497

[11] Kos‐Kudła B , Castaño JP , Denecke T , et al. European Neuroendocrine Tumour Society (ENETS) 2023 guidance paper for nonfunctioning pancreatic neuroendocrine tumours. J Neuroendocrinol. 2023;35(12):e13343. doi:10.1111/jne.13343 37877341

[12] Lamarca A , Bartsch DK , Caplin M , et al. European neuroendocrine tumor society (ENETS) 2024 guidance paper for the management of well‐differentiated small intestine neuroendocrine tumours. J Neuroendocrinol. 2024;36(9):e13423. doi:10.1111/jne.13423 38977327

[13] Fazio N , La Salvia A . Precision medicine in gastroenteropancreatic neuroendocrine neoplasms: where are we in 2023? Best Pract Res Clin Endocrinol Metab. 2023;37(5):101794. doi:10.1016/j.beem.2023.101794 37414651

[14] Kazmierczak PM , Rominger A , Wenter V , et al. The added value of 68Ga‐DOTA‐TATE‐PET to contrast‐enhanced CT for primary site detection in CUP of neuroendocrine origin. Eur Radiol. 2017;27(4):1676‐1684. doi:10.1007/s00330-016-4475-3 27436022

[15] De Dosso S , Treglia G , Pascale M , et al. Detection rate of unknown primary tumour by using somatostatin receptor PET/CT in patients with metastatic neuroendocrine tumours: a meta‐analysis. Endocrine. 2019;64(3):456‐468. doi:10.1007/s12020-019-01934-9 31004334

[16] Modlin IM , Oberg K , Chung DC , et al. Gastroenteropancreatic neuroendocrine tumours. Lancet Oncol. 2008;9:61‐72. 18177818 10.1016/S1470-2045(07)70410-2

[17] Padwal MK , Parghane RV , Chakraborty A , et al. Developing a peripheral blood RNA ‐seq based NETseq ensemble classifier: a potential novel tool for non‐invasive detection and treatment response assessment in neuroendocrine tumor patients receiving ^177^Lu—DOTATATE PRRT. J Neuroendocrinol. 2025;37(3):e13462. doi:10.1111/jne.13462 39539072 PMC11919474

[18] Blazevic A , Starmans MPA , Brabander T , et al. Predicting symptomatic mesenteric mass in small intestinal neuroendocrine tumors using radiomics. Endocr Relat Cancer. 2021;28(8):529‐539. doi:10.1530/ERC-21-0064 34003139

[19] von Stempel C , Blazevic A , Starmans M , et al. Validation of a radiomics model to predict symptoms complications from small intestinal NET mesenteric metastases‐preliminary report. J Neuroendocrinol. 2024;36:196.

[20] Lopez‐Ramirez F , Soleimani S , Azadi JR , et al. Radiomics machine learning algorithm facilitates detection of small pancreatic neuroendocrine tumors on CT. Diagn Interv Imaging. 2025;106(1):28‐40. doi:10.1016/j.diii.2024.08.003 39278763

[21] Zheng K , Duan J , Wang R , et al. Deep learning model with pathological knowledge for detection of colorectal neuroendocrine tumor. Cell Rep Med. 2024;5(10):101785. doi:10.1016/j.xcrm.2024.101785 39413732 PMC11513840

[22] Williams ED , Sandler M . The classification of carcinoid tumours. Lancet. 1963;281(7275):238‐239. doi:10.1016/S0140-6736(63)90951-6 14000847

[23] Redemann J , Schultz FA , Martinez C , et al. Comparing deep learning and immunohistochemistry in determining the site of origin for well‐differentiated neuroendocrine tumors. J Pathol Inform. 2020;11(1):32. doi:10.4103/jpi.jpi_37_20 33343993 PMC7737494

[24] Bankhead P , Loughrey MB , Fernández JA , et al. QuPath: open source software for digital pathology image analysis. Sci Rep. 2017;7(1):16878. doi:10.1038/s41598-017-17204-5 29203879 PMC5715110

[25] Mormont R , Geurts P , Marée R . Comparison of deep transfer learning strategies for digital pathology. 2018 IEEE/CVF Conference on Computer Vision and Pattern Recognition Workshops (CVPRW). IEEE; 2018:2343–2384. doi:10.1109/CVPRW.2018.00303

[26] Wang X , Du Y , Yang S , et al. RetCCL: clustering‐guided contrastive learning for whole‐slide image retrieval. Med Image Anal. 2023;83:102645. doi:10.1016/j.media.2022.102645 36270093

[27] Kang Y , Kim YJ , Park S , et al. Development and operation of a digital platform for sharing pathology image data. BMC Med Inform Decis Mak. 2021;21(1):114. doi:10.1186/s12911-021-01466-1 33812383 PMC8019341

